# *LHPP* suppresses colorectal cancer cell migration and invasion in vitro and in vivo by inhibiting Smad3 phosphorylation in the TGF-β pathway

**DOI:** 10.1038/s41420-021-00657-z

**Published:** 2021-10-04

**Authors:** Bin Hou, Wenhan Li, Peng Xia, Fengyu Zhao, Zhao Liu, Qingnuo Zeng, Shilong Wang, Dongmin Chang

**Affiliations:** 1 Department of Thoracic Surgery, Shaanxi Provincial People’s Hospital, 256W, Youyi Road, Xi’an, 710068 Shaanxi China; 2 Department of Surgical Oncology, Shaanxi Provincial People’s Hospital, 256W, Youyi Road, Xi’an, 710068 Shaanxi China; 3 Department of Surgical Oncology, the First Affiliated Hospital of Xi’an Jiaotong University, 277W, Yanta Road, Xi’an, 710061 Shaanxi China

**Keywords:** Colorectal cancer, Epithelial-mesenchymal transition

## Abstract

The roles of phospholysine phosphohistidine inorganic pyrophosphate phosphatase (*LHPP*) in tumorigenesis have been recently proven in hepatocellular carcinoma (HCC), cervical, pancreatic, bladder, and thyroid cancers. Previous research demonstrated that *LHPP* repressed cell proliferation and growth by inactivating the phosphatidylinositol 3-kinase/*AKT* signaling pathway in vitro and in vivo. However, the functions and potential mechanisms of *LHPP* as a tumor suppressor in colorectal cancer (CRC) metastasis are still unknown. Consequently, the Transwell assay and xenograft nude model showed that *LHPP* inhibited migration and invasion of CRC cells in vitro and in vivo, respectively. The expression of total and nuclear epithelial-to-mesenchymal transition (EMT)-related proteins were significantly reduced after *LHPP* upregulation. Human Gene Expression Array and IPA (Ingenuity Pathway Analysis) commercial software were applied to identify differentially expressed genes (DEGs) and potential cell signaling pathways. A total of 330 different genes were observed, including 177 upregulated genes and 153 downregulated genes. Bioinformatics analysis suggested that the transforming growth factor-β (TGF-β) signaling pathway was highly inactivated in this study. Then, Smad3 phosphorylation was apparently decreased, whereas Smad7 expression was markedly enhanced after upregulating *LHPP* expression. These results were proven once again after TGF-β1 stimulation. Furthermore, a specific inhibitor of Smad3 phosphorylation (SIS3) was applied to verify that *LHPP* repressed EMT of cancer cells by attenuating TGF-β/Smad signaling. The results suggested that suppression of the TGF-β/Smad signaling pathway by *LHPP* overexpression could be abolished by SIS3.

## Introduction

Colorectal cancer (CRC) is one of the most malignant gastrointestinal cancers worldwide. Although the prognosis of patients with CRC has improved due to recent developments in colonoscopy, a 5-year relative survival still remains less than 50% in low-income countries [1, 2]. The situation in China has become more serious. The estimated new CRC cases and deaths of colorectal cancer in 2015 were 376,300 and 191,000, respectively [3]. The number of patients with CRC < 45 years old was surprisingly high (25,300) [3]. Thus, it is urgent to uncover the pathological mechanism of colorectal cancer to develop new therapy.

CRC development is a multistep process with different gene mutations, such as *APC*, *TP53*, *RAS*, and *EGFR*, promoting tumor proliferation, invasion, and metastasis [4–6]. In most cases, cancer metastasis and recurrence are considered as the first reasons for the high rate of mortality and poor prognosis of patients. Epithelial-to‐mesenchymal transition (EMT) is the most important part of cell invasion and migration and is also highly associated with drug resistance, cancer stemness, and apoptosis [7, 8]. Cells lose intracellular tight junctions and contacts and acquire mesenchymal traits after EMT. Many signaling pathways [9, 10] are involved in this phenomenon, including transforming growth factor-β (TGF-β), Wnt/β-catenin, Notch, and nuclear factor-B (NF-ĸB). Recently, increasing studies [11] have proven that phosphatidylinositol 3-kinase (PI3K)/AKT also promoted EMT development. The activation of these signaling pathways mediates downstream crucial molecules. E-cadherin protein downregulation and N-cadherin protein upregulation are the most significant step during EMT. E-cadherin, an essential cell-to-cell adhesion protein, forms epithelial cell sheets and maintains cell quiescence. Conversely, increasing N-cadherin expression is normally observed in migrating neurons and mesenchymal cells. Other pivotal biomarkers, including Snail, Slug, Twist1, and Zeb1/2, could also regulate the EMT process during embryogenesis or tumorigenesis by decreasing E-cadherin protein expression or enhancing N-cadherin protein expression directly or indirectly. Nowadays, increasing evidence suggested that exosomes, extracellular vehicles 30–150 nm in size, could also mediate EMT in different cancers [12–14].

Phospholysine phosphohistidine inorganic pyrophosphate phosphatase *(LHPP)*, a type of histidine phosphatase protein, has been proven as a tumor suppressor in hepatocellular carcinoma [15] (HCC), cervical [16], pancreatic [17], bladder [18], and thyroid cancers [19] by regulating the AKT signaling pathway. In the previous research [20], LHPP protein overexpression was highly related to better prognosis and lower tumor–node–metastasis classification of patients with CRC. *LHPP* could inhibit colorectal cancer cell proliferation and growth by inactivating the PI3K/AKT signaling pathway. This conclusion was consistent with previous studies. Interestingly, Tian et al. [21] predicted that miR-363-5p and miR-765 could be potential molecules to target *LHPP* expression in AFP-negative HCC patients, providing researchers a new perspective to investigate *LHPP* mechanisms in tumorigenesis.

This study attempted to further uncover the biological functions of LHPP protein in CRC metastasis. First, another CRC cell line (Caco2) was used to verify the conclusions of a previous study. Whether *LHPP* overexpression could repress CRC cell metastasis was also investigated in vivo and in vitro. Furthermore, *LHPP* was identified as a target gene to inhibit the TGF-β signaling pathway by repressing Smad3 phosphorylation.

## Results

### *LHPP* gene definitely inhibits CRC cell growth

To verify previous conclusions, *LHPP-*overexpressing LVs were transferred to Caco2 and short hair-cap LVs to Sw480, respectively. The results are shown in Supplemental Fig.1 (Sw480 *LHPP*-overexpression and HT-29 *Sh-LHPP* cell lines have been constructed before [20]). As predicted, compared to the negative group, the cell viability of the *OE-LHPP* group was significantly decreased (*P* < 0.001; Supplemental Fig. 2a) in Caco2 cells at 3, 5, and 7 days after transfection with *OE-LHPP* LV. Clone numbers were also apparently reduced in the *OE-LHPP* group (all *P* < 0.001, Supplemental Fig. 2c, e). Conversely, downregulating LHPP protein expression could promote CRC cells proliferation. The viability of Sw480 cells was markedly higher in the *Sh-LHPP* group at 3, 5, and 7 days after transferring short hair-cap LVs (all *P* < 0.01, shown in Supplemental Fig. 2b). Clone formation experiments also suggested that blocking *LHPP* expression had more clone numbers in the *Sh-LHPP* group (*P* < 0.0001, Supplemental Fig. 2d, f).

**Fig. 1:**
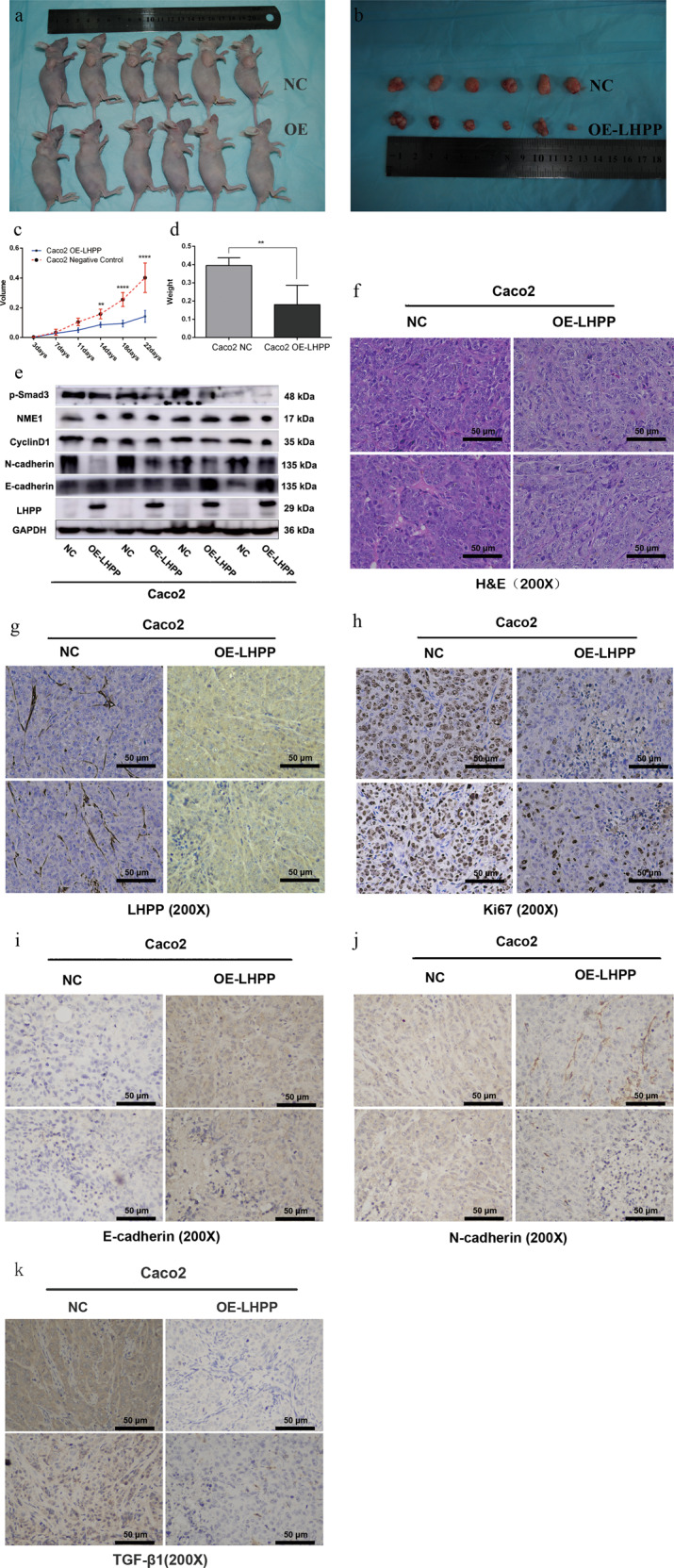
*LHPP* inhibits the tumor formation of colorectal cancer cells in vivo. **a**, **b** Typical images of nude mouse model after injecting with Caco2 OE-LHPP cells and negative control cells. **c**, **d** The Graphpad Prism 6 software was used to analyze the weight and volume of tumors in different groups (g in weight and mm^3^ in volume). The tumors in the negative group grew much larger and heavier than tumors in the OE-LHPP group. **e** The proteins were isolated from tumors and analyzed via western blot. Expression of EMT-related markers, such as N-cadherin, p-Smad3, were downregulated in the Caco2 OE-LHPP group. **f** Tumors’ tissue was stained by hematoxylin and eosin (H&E, scale bar 50 μm, ×200). **g** Expression of LHPP protein was examined by using IHC (scale bar 50 μm, ×200). **h** The biomarker of cell viability, Ki-67, was determined by using IHC (scale bar 50 μm, ×200). **i** Expression levels of E-cadherin in negative control and experimental groups were evaluated using IHC (scale bar 50 μm, ×200). **j** The biomarker of EMT, N-cadherin protein, was tested using IHC (scale bar 50 μm, ×200). **k** TGF-β1 protein expression was examined by using IHC (scale bar 50 μm, ×200). **P < 0.01, ***P < 0.001, ****P < 0.0001.

To examine the influence of LHPP protein on tumor progression in vivo, Caco2 cells after transferring *OE-LHPP* LVs were injected into female nude mice. Each group contained six mice. Notably, tumors in the *OE-LHPP* group developed much slower and lighter than the NC group (all *P* < 0.05, Fig. 1a–d). The mean weight of tumors in the experimental and NC groups were 0.1800 ± 0.0435 and 0.3950 ± 0.0175 g, respectively. Furthermore, tumor tissues were confirmed by using hematoxylin and eosin (H&E) staining (Fig. 1f). The proportion of Ki-67, a specific biomarker of cell proliferation, was determined using IHC. In Fig. 1h, the Ki-67-staining score was significantly higher in the NC group than in the *LHPP* overexpression group. Tumors and extracted total proteins were isolated to evaluate the expression of cell growth biomarkers. Consequently, cyclinD1 and NME1 expression was apparently decreased in the *LHPP-*overexpressing group (Fig. 1e). The expression levels of EMT-related proteins extracted from the xenograft tumor tissues via western blot analysis and IHC were evaluated. E-cadherin protein expression levels were enhanced and N-cadherin was reduced in the Caco2 *OE-LHPP* group (Fig. 1e, i, j). Consistently, TGF-β1 expression was higher in the Caco2 NC group compared to the *OE-LHPP* group using IHC assay (Fig. 1k).

### *LHPP* regulated CRC cell proliferation by suppressing the transition from the G_0_/G_1_ to M phase

In Supplemental Fig. 3a, b, FCM indicated that upregulating *LHPP* expression in Caco2 cells reduced the proportion of S-phase cells. Proteins that were involved in regulating the cell cycle, including p53, cyclinD1, CDK4, PCNA, and NME1, had a significantly positive difference between Caco2 NC and *OE-LHPP* groups (Supplemental Fig. 3e, f). *LHPP* impeded the expression of cyclinD1/CDK4, NME1, and PCNA expression and promoted p53 expression. In addition, *LHPP* silencing demonstrated a remarkable reduction in the G_0_/G_1_ phase and an enhancement in the S-phase (Supplemental Fig. 3c, d). CyclinD1, NME1, and PCNA expression were elevated after *LHPP* depletion (Supplemental Fig. 3e, f). Consistently, *LHPP* knockdown decreased p53 expression. These results suggested that *LHPP* could mediate the cell cycle by downregulating cyclinD1/CDK4 expression and upregulating p53 expression.

### *LHPP* suppressed CRC migration and invasion in vitro and in vivo

**Fig. 2:**
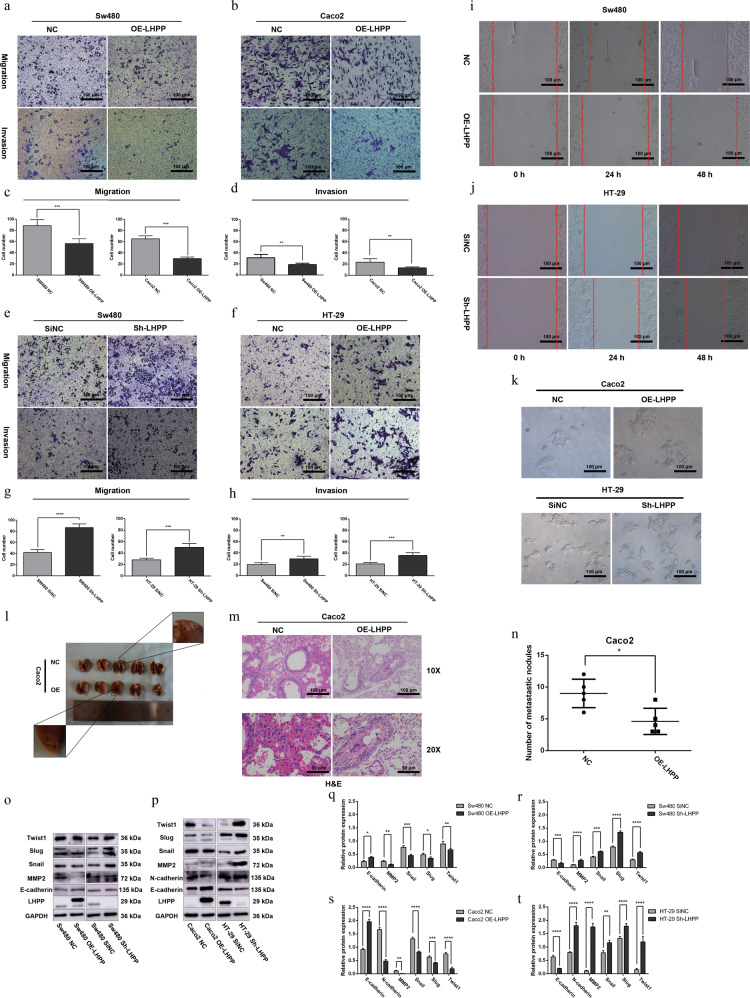
*LHPP* restains metastatic ability of colorectal cancer cells in vivo and vitro. Effect of LHPP overexpression on the migration and invasion (matrix gel 50–80 μl, dilution 1:8, 8-μm pore) of Sw480 (**a**) and Caco2 (**b**) cell lines compared to their corresponding control groups (scale bar, 100 μm, ×100). The quantification of migration and invasion in Sw480 cells (**c**) and Caco2 (**d**) were calculated using the Graphpad Prism 6 software according to Transwell assay, Migrated and invasive cells were stained and counted in at least five microscope fields. Blocking LHPP increased migration and invasion abilities of (matrix gel 50–80 μl, dilution 1:8, 8-μm pore) Sw480 (**e**) and HT-29 (**f**) cells when compared to negative groups (scale bar, 100 μm, ×100); The statistical differences were calculated by the Graphpad Prism 6 software HT-29 (**g**) and Sw480 (**h**). Migrated and invasive cells were stained and counted in at least five microscope fields. **i** Effect of LHPP overexpression on the migration of Sw480 cells at 24 and 48 h (scale bar 100 μm, ×100). **j** LHPP downregulation promoted HT-29 cells migration determined by wound-healing assay at 24 and 48 h (scale bar 100 μm, ×100). **k** The change of Caco2 cells’ shape following enhancement of LHPP expression; The change of HT-29 cells’ shape after reduction of LHPP expression (scale bar, 100 μm, ×100). **l** The representative images of lungs from nude mice (*n* = 5), black arrows showed tumor metastasis. **m** Lung nodules were identified using H&E staining. **n** Average number of tumor nodules in lung metastasis in Caco2 OE-LHPP group and their control group. **o** EMT-related proteins including E-cadherin, MMP2, Snail, Slug, and Twist1 were evaluated by using western blot in Sw480 cells after upregulating and downregulating LHPP expression. **p** Protein levels of E-cadherin, N-cadherin, MMP2, Snail, Slug, and Twist1 were tested using Western blot assay in Caco2 with high expression of LHPP and HT-29 with low expression of LHPP. **q**–**t** The Graphpad Prism 6 and Image J software were applied to observe the statistical differences between the experimental group and their control group. *P < 0.05, **P < 0.01, ***P < 0.001, ****P < 0.0001.

To investigate whether *LHPP* could influence migration and invasion abilities of CRC cells, wound-healing assay and Transwell assay with or without the Matrigel matrix layer in the chambers (50–100 μl matrix gel per well, diluted 1:8, dilution with medium). Increasing *LHPP* expression apparently attenuated CRC migration and invasion abilities compared to their corresponding groups (all *P* < 0.05, Fig. 2a–d). Consistent with the results above, *LHPP-*depletion increased cell numbers in the Sw480 and HT-29 *Sh-LHPP* groups compared to NC groups (Fig. 2e, f). There were significant differences between experimental and NC groups (all *P* < 0.01, Fig. 2g, h). The results of the wound-healing assay showed the blank area in Sw480 overexpression groups was larger than the blank area in the control groups at 24 and 48 h (Fig. 2i). While knockdown *LHPP* expression promoted HT-29 cells wound-healing rate at 24 and 48 h (Fig. 2j). A microscope was used to observe cell shape alternation. In Fig. 2k, HT-29 cells gradually loosened intracellular tight junctions between cells after reducing *LHPP* expression, suggesting that HT-29 acquired more mesenchymal traits. Conversely, upregulating *LHPP* expression could reverse the mesenchymal features of Caco2 cells and retain cell epithelial integrity.

To further determine whether *LHPP* affected CRC cell migration in vivo, Caco2 cells were injected into BALB/c-nude mice through the tail vein with stable *LHPP* overexpression. Lung metastatic nodules were counted under microscopy by H&E staining (Fig. 2l, m). The Number of metastatic nodules in *OE-LHPP* was lesser than in the NC group (Fig. 2n, *P* < 0.05).

### *LHPP* repressed CRC cell EMT and influenced matrix metalloproteinase (MMP) levels

The EMT is a complex process that involves the activation of many molecules, including E-cadherin, N-cadherin, Slug, Snail, and Twist1/2. Therefore, western blot assay was used to determine the expressions of these proteins. Increasing *LHPP* expression evidently caused the suppression of N-cadherin, Slug, Snail, and Twist1 in Sw480 and Caco2 cells and the significant promotion of E-cadherin (Fig. 2o, p, q, s; all *P* < 0.05). *LHPP* depletion produced opposite results. E-cadherin expression was much lower in Sw480 and HT-29 *Sh-LHPP* cells than in their counterparts (Fig. 2o, p, r, t; all *P* < 0.001). The other key components of EMT, such as N-cadherin, Slug, Snail, and Twist1, were highly activated in Sw480 and HT-29 cells after knockdown *LHPP* expression.

Generally, MMP families disrupted the basal lamina and invaded the epithelial layer up to the deep layer. Among them, MMP2 and MMP9 played a crucial role in this process. Thus, MMP2 expression was evaluated in different CRC cell lines. As expected, compared to negative groups, *LHPP* Overexpression positively repressed the MMP2 protein level in Sw480 and Caco2 cell lines (Fig. 2o, p, *P* < 0.01). In contrast, MMP2 were upregulated in Sw480 and HT-29 *LHPP*-depletion groups (Fig. 2o, p, *P* < 0.01). Accordingly, these above results revealed that *LHPP* could negatively mediate EMT-related proteins and suppress CRC metastasis.

### *LHPP* influenced cancer cell migration and invasion by mediating the TGF-β/Smad signaling pathway

**Fig. 3:**
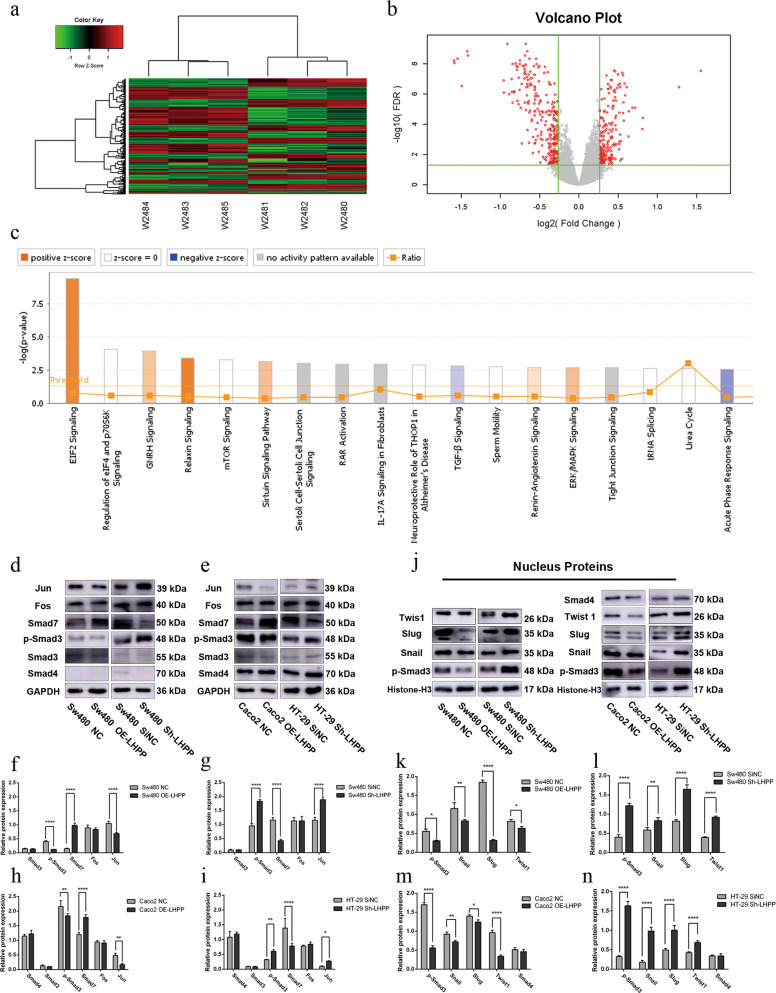
Results of human microarray and Ingenuity pathway analysis (IPA), Caco2 NC groups: W2480–2482, Caco2 OE-LHPP groups: W2483–2485. **a** Hierarchical clustering of genes which were differentially expressed in Caco2 LHPP overexpression group and negative control group; The cut-off values were | Fold change | ≥1.2 and FDR < 0.05. **b** Volcano plot: red points mean genes with statistical difference at least 1.2-fold change and FDR < 0.05. **c** Ingenuity pathway analysis. The orange rectangle means activated cell pathway and the blue rectangle means inactivated cell pathway. Positive *z* score >2.0 was regarded as highly activated. Conversely, a negative *z* score < −2.0 was regarded as highly inhibited; TGF-β/Smad signaling pathway was inactivated after upregulating the expression of LHPP in the Ccaco2 cell line. Pivotal molecules of TGF-β/Smad signaling pathway, including Smad3, p-Smad3, Smad4, Smad7, Jun, and Fos, were evaluated in Sw480 (**d**) and Caco2 and HT-29 cell lines (**e**) using western blot assay. **f**–**i** Relative expression levels of these proteins were calculated using Image J and Graphpad Prism 6 software. **j** Cell nuclear proteins, such as p-Smad3, Smad4, Snail, Slug, and Twist1 were also examined using western blot, Histon-H3 was served as control. The quantification results were presented in **k** to **n**. *P < 0.05, **P < 0.01, ***P < 0.001, ****P < 0.0001.

The mechanism of *LHPP*-mediated tumorigenicity was further investigated by microarray and IPA. Hierarchical clustering analysis demonstrated that *LHPP* overexpression led to a significant gene expression profile with 177 upregulated genes and 153 downregulated genes (Fig. 3a, b and Table 1). IPA showed that various signaling pathways were involved in CRC development after *LHPP* overexpression (Fig. 3c and Table 2). Among them, the TGF-β signaling pathway attracted the authors’ attention as it was highly correlated with EMT and consistent with previous observation. The expression level of pivotal molecules in the TGF-β signaling pathway was examined. The results showed that *LHPP* overexpression apparently caused a reduction of p-Smad3 protein and a significant increase of Smad7 which was a negative regulator in the TGF-β/Smad signaling pathway (Fig. 3d–i, all *P* value <0.01). These conclusions were reversed after *LHPP* knockdown in Sw480 and HT-29 cell lines (Fig. 3d–i, all *P* value <0.01). There was no significant difference observed in Fos and Smad4 protein expression levels between the experimental groups and NC groups.

**Table 1:** A part of differentially expressed genes is shown.

Gene symbol	Gene title	Fold change	Regulation	*P* value	FDR
PBDC1	Polysaccharide biosynthesis domain containing 1	2.938683526	Up	2.21892E-11	3.01077E-08
EIF5	Eukaryotic translation initiation factor 5	1.644706322	Up	2.8655E-08	8.23027E-06
JUN	Jun proto-oncogene	1.526220419	Up	6.66257E-10	3.54278E-07
FOS	FBJ murine osteosarcoma viral oncogene homolog	1.497362297	Up	2.92128E-10	2.01779E-07
WNT5A	Wingless-type MMTV integration site family member 5A	1.463473545	Up	0.000495577	0.019657741
ING3	Inhibitor of growth family member 3	1.42711672	Up	6.86333E-05	0.004704966
APP	Amyloid beta (A4) precursor protein	1.413609199	Up	4.41865E-10	2.59507E-07
SAMD5	Sterile alpha motif domain containing 5	1.389134861	Up	7.74637E-09	2.80141E-06
DAPK1	Death-associated protein kinase 1	1.348217572	Up	3.25821E-08	9.15767E-06
SAMD9	Sterile alpha motif domain containing 9	1.31141412	Up	4.62297E-07	8.87361E-05
RAP1A	RAP1A, member of RAS oncogene family	−3.007177445	Down	1.64814E-12	6.14637E-09
ADGRF1	Adhesion G protein-coupled receptor F1	−2.808240835	Down	5.2499E-10	3.03792E-07
CEP19	Centrosomal protein 19 kDa	−1.942906759	Down	1.72551E-10	1.28108E-07
PPP1R2	Protein phosphatase 1 regulatory inhibitor subunit 2	−1.718436649	Down	9.95258E-12	1.63177E-08
ZNF175	Zinc finger protein 175	−1.579480362	Down	3.05502E-09	1.2393E-06
TGFB2	Transforming growth factor beta 2	−1.497853647	Down	5.9866E-06	0.000701044
NRROS	Negative regulator of reactive oxygen species	−1.375382918	Down	0.000321363	0.014484906
BCL6	B-cell CLL/lymphoma 6	−1.304231528	Down	2.08752E-05	0.00189601
VIM	Vimentin	−1.282252159	Down	0.000246768	0.011899609
PRKD3	Protein kinase D3	−1.269505519	Down	9.61151E-05	0.006166303

The differentially expressed genes were analyzed: Human Gene Expression Array and IPA analysis after overexpressing *LHPP* in Caco2 cells compared to the corresponding group. Significant difference was |fold change | ≥1.2 and FDR < 0.05 (*P* < 0.05). “+” means this gene was upregulated and “−” means this gene was downregulated.

**Table 2:** Canonical signaling pathways are shown.

Ingenuity canonical pathways	−log(*P* value)	*z* score
EIF2 signaling	9.4	1.265
Relaxin signaling	3.39	1.342
ERK/MAPK signaling	2.68	0.707
BMP signaling pathway	2.28	0.447
PPARα/RXRα activation	1.37	1.633
TGF-β signaling	2.76	−0.447
Acute-phase response signaling	2.53	−0.816
SPINK1 pancreatic cancer pathway	2.04	−2
PPAR signaling	1.91	−0.447
Glioma invasiveness signaling	1.66	−1

The canonical signaling pathways were analyzed using IPA software based on the results of differentially expressed genes. ten was the base of −log(*P* value). −Log(*P* = 0.05) = 1.3, Therefore, the statistical significance was −log(*P* value)>1.3. *z* score >0 represented this signaling pathway was activated in the research; *z* score <0 represented this signaling pathway was inhibited in this experiment. The more scores the signaling pathway got in the study, the more obvious the statistical difference was. The results provided us potential materials to investigate the relationship between *LHPP* biological functions and classical signaling pathways.

The expression level of nuclear proteins, such as p-Smad3, Snail, Slug, Twist1, and Smad4, was also determined. *LHPP* suppressed Smad3 phosphorylation, leading to the obstruction of EMT-related proteins transcription (Fig. 3j–n). This finding was also proven after blocking *LHPP* expression (Fig. 3j–n). These data suggested that *LHPP* probably inhibited CRC migration and invasion by repressing Smad3 phosphorylation and upregulating Smad7 expression level.

### Silenced *LHPP* had no contribution to the apoptotic rate of CRC cells

Based on previous references, the Eif2 signaling pathway was highly associated with cell apoptosis. Subsequently, whether knockdown of *LHPP* expression influenced cell apoptosis was evaluated by 7-AAD/PE staining and fluorescence-activated cell sorting analysis. In Supplemental Fig. 4a–d, there was no significant difference observed between experimental groups and their counterparts. Furthermore, the key regulators in cell apoptosis and the Eif2 signaling pathway, including Bax, caspase3, Eif2, and p-Eif2, did not change after blocking *LHPP* expression (Supplemental Fig. 4e, f).

### *LHPP* definitely repressed TGF-β-induced EMT and metastasis ability of cancer cells

**Fig. 4:**
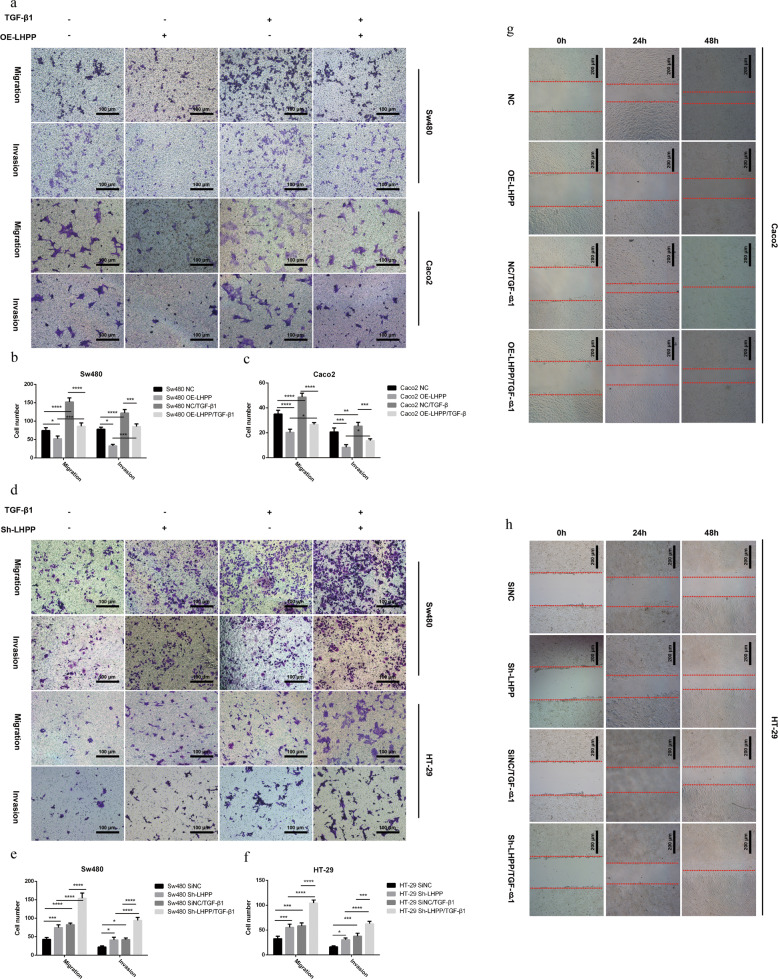
The Transwell assay was performed to test migration and invasion (matrix gel 50 μl, dilution 1:8, 8-μm pore, scale bar, 100 μm, ×100) abilities of colorectal cancer cells after TGF-β1 stimulation (5 ng/ml) for 24 h. **a** Effect of LHPP overexpression on migration and invasion abilities of Sw480 and Caco2 cells. **b**, **c** The quantification results were calculated using the Graphpad Prism 6 software. Migration and invasion cells were stained and counted in at least five microscope fields. **d** Silencing of LHPP expression promoted TGF-β1-induced migration and invasion abilities in Sw480 and HT-29 cells. **e**, **f** The significant difference was determined using the Graphpad Prism 6 software. Migration and invasion cells were stained and counted in at least five microscope fields. **g**, **h** We used wound-healing assay to verify the migration capability of Caco2 and HT-29 cell line after treating with TGF-β1. The scratch was observed at 0, 24, and 48 h using microscope (scale bar, 200 μm, ×40); *P < 0.05, **P < 0.01, ***P < 0.001, ****P < 0.0001.

To further support that the TGF-β signaling pathway was involved in the mechanism of *LHPP*-mediated tumorigenesis, cancer cells were treated with TGF-β1 (5 ng/ml), the most powerful EMT inducer, after being serum starvation for 24 h. After TGF-β1 stimulation for 24 h, compared to the NC group, the migration and invasion abilities of Sw480 and Caco2 (Fig. 4a) with stable *LHPP* overexpression were also significantly impaired. Statistical results are shown in Fig. 4b, c, respectively. Conversely, silencing LHPP expression enhanced the metastatic ability of Sw480 and HT-29 cells (Fig. 4d) after TGF-β1 stimulation for 24 h. Statistical results were shown in Fig. 4e, f, respectively. Moreover, the wound-healing assay indicated *LHPP*-overexpressing Caco2 had more blank area in comparison to the corresponding group after TGF-β1 stimulation 24 h (Fig. 4g). Blocking expression of *LHPP* in HT-29 cells led to a stronger healing ability (Fig. 4h) following TGF-β1 stimulation.

**Fig. 5:**
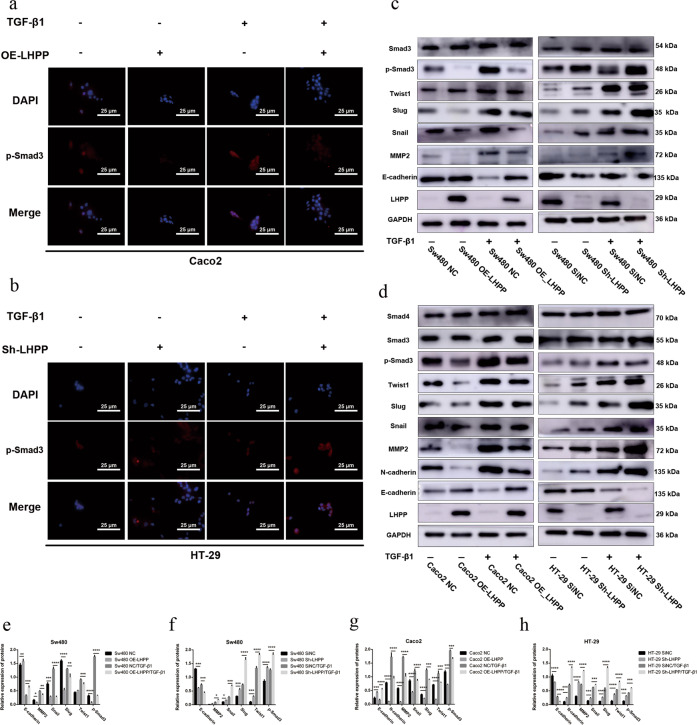
*LHPP* blocks expression levels of key proteins in canonical TGF-β/Smad pathway. **a**, **b** Expression level of p-Smad3 was evaluated by using immunofluorescence assay after TGF-β1 stimulation for 24 h, cell nuclei were counterstained with DAPI and observed under a fluorescence microscope (scale bar: 25 μm). **c**, **d** In response to TGF-β1 stimulation, key molecules involved in the TGF-β/Smad classical signaling pathway were determined using western blot assay. **e**, **f** Relative expression levels of these proteins were calculated using Image J and Graphpad Prism 6 software. *P < 0.05, **P < 0.01, ***P < 0.001, ****P < 0.0001.

Furthermore, the IF assay was performed to determine the p-Smad3 protein level after TGF-β1 stimulation. Consistent with these observations, the IF signal of p-Smad3 was obviously impaired in Caco2 cells with stable upregulation of LHPP (Fig. 5a). In contrast, LHPP knockdown apparently enhanced TGF-β-induced Smad3 phosphorylation in HT-29 cells (Fig. 5b). These results indicated LHPP certainly weakened TGF-β-induced CRC metastasis by mediating Samd3 phosphorylation in the TGF-β signaling pathway.

With TGF-β1 activation, the expression level of EMT-related markers involved in the TGF-β signaling pathway was examined. In Fig. 5c, d, E-cadherin expression was remarkably increased in Sw480 and Caco2 cell lines with stable *LHPP* overexpression compared to NC groups after TGF-β1 stimulation. Data showed an apparent reduction in the expression levels of EMT-related proteins, including N-cadherin, MMP2, Snail, Slug, and Twist1. Statistical results are shown in Fig. 5e, g. Notably, this phenomenon was reversed by downregulating LHPP expression. In response to TGF-β-induced EMT, the HT-29-Sh-LHPP and Sw480-Sh-LHPP groups demonstrated obviously higher expression levels of N-cadherin, MMP2, Snail, Slug, and Twist1 but a lower expression level of E-cadherin (Fig. 5c, d). Statistical results are shown in Fig. 5e, g. As for the crucial points involved in the TGF-β/Smad signaling pathway, the p-Smad3 protein level was also suppressed in Sw480 and Caco2 *OE-LHPP* groups after TGF-β1 activation compared to NC groups. The opposite result was observed in LHPP knockdown cells. Smad3 phosphorylation was highly activated after TGF-β1 stimulation in HT-29 and Sw480 cell lines. This result was supported by the IF assay mentioned previously (Fig. 5a, b).

**Fig. 6:**
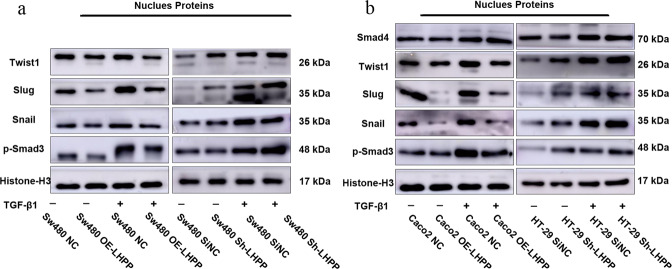
We extracted nuclear proteins from different colorectal cancer cell lines after TGF-β1 treatment for 24 h. **a**, **b** Expression levels of EMT-related transcription factors in colorectal cancer cells were examined by using western blot assay after changing LHPP expression. Histone-H3 protein was regarded as the control.

Furthermore, cell nuclear proteins, including p-Smad3, Snail, Slug, and Twist1, were also determined after TGF-β1 stimulation for 24 h. As expected, the expression levels of p-Smad3, Snail, Slug, and Twist1 were markedly decreased in Sw480 and Caco2 cells (Fig. 6). Conversely, blocking LHPP significantly promoted the expression of nuclear proteins, such as p-Smad3, Snail, Slug, and Twist1, in HT-29 and Sw480 cells compared to their counterparts.

### Suppression of the TGF-β/Smad signaling pathway by *LHPP* overexpression could be abolished by SIS3, a specific inhibitor of Smad3 phosphorylation, in CRC cells

The above results suggested that LHPP could impair TGF-β-mediated EMT and metastatic cell ability by inactivating the TGF-β/Smad signaling pathway, whereas other non-Smad canonical signaling pathways, such as the mitogen-activated protein kinase (MAPK) pathway, might also be involved in TGF-β-induced EMT. To investigate the specificity of the TGF-β/Smad pathway in CRC progression, Sw480, Caco2, and HT-29 cells were pretreated with different concentrations of SIS3 for ~4–6 h. Subsequently, TGF-β1 (5 ng/ml) was added into the medium after serum starvation for 24 h. In Supplemental Fig. 5, SIS3 markedly reduced the expression level of p-Smad3 in Sw480 (3 ng/ml) and Caco2 (5 ng/ml) cells. Besides, HT-29 cells treated with 10 ng/ml SIS3 showed a significant decrease in expression of p-Smad3. Therefore, we chose 3 ng/ml, 5 ng/ml, and 10 ng/ml SIS3 to block phosphorylation of Smad3 in Sw480, Caco2, and HT-29 cell lines, respectively.

**Fig. 7:**
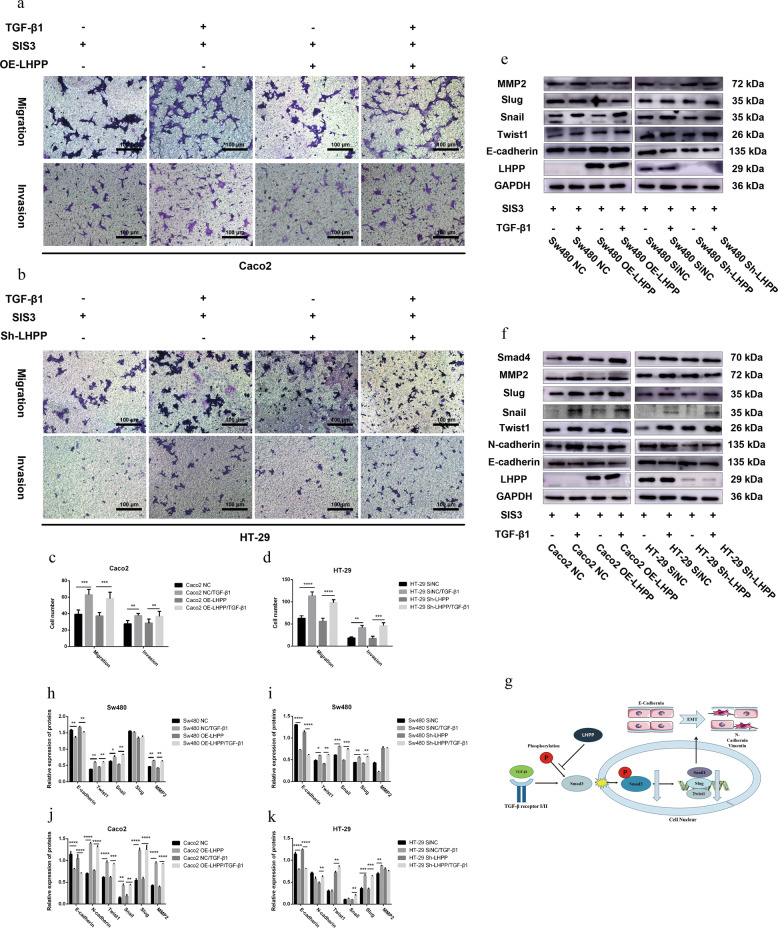
In order to verify whether LHPP could inhibit migration and invasion abilities of colorectal cancer cells via inactivating phosphorylation of Smad3 specifically or not. **a**, **b** The Transwell assay was used for evaluating the influence of SIS3 (details described before) on TGF-β1-mediated cell migration and invasion abilities (matrix gel 80 μl, dilution 1:8, 8-μm pore, scale bar, 100 μm, ×100). **c**, **d** Quantification results were determined using the Graphpad Prism 6 software. Migrated and invasive cells were stained and counted in at least five microscope fields. **e**, **f** Expression levels of EMT-related proteins in colorectal cancer cell lines. A significant difference could be observed not only in the overexpression group but also in the downexpression group with or without TGF-β1 treatment. **g** Potential mechanism of LHPP in migration and invasion abilities of the colorectal cancer cell. **h**–**k** Relative expression levels of biomarkers were calculated using Image J and Graphpad Prism 6 software. *P < 0.05, **P < 0.01, ***P < 0.001, ****P < 0.0001.

SIS3 was administered to block the TGF-β/Smad signaling pathway in LHPP upregulation and downregulation groups. Consequently, SIS3 treatment could abolish changes of EMT-related protein levels, such as E-cadherin, N-cadherin, Twist1, Snail, Slug, and MMP2, in LHPP-overexpressing Sw480 and Caco2 cells (Fig. 7e, f) compared to the NC group. These were proved not only in TGF-β1-positive groups but also in TGF-β1-negative groups. Statistical results are shown in Fig. 7h, j, respectively. Consistently, SIS3 also significantly abolished EMT-related protein variation in LHPP knockdown HT-29 and Sw480 cells treated with or without 5 ng/ml TGF-β1 (Fig. 7e and f). Statistical results are shown in Fig. 7i, k, respectively. The above results were proven again by using the Transwell assay in Caco2 LHPP upregulation (Fig. 7a) and HT-29 LHPP downregulation cells (Fig. 7b) with SIS3 pretreatment. There was no significant difference between experimental and negative control groups (Fig. 7c, d). When CRC cells treated with SIS3, LHPP could not play its role in the progression of colorectal cancer. These data indicated that LHPP did repress the TGF-β/Smad signaling pathway by inhibiting Smad3 phosphorylation.

## Discussion

*LHPP*, a 29 kDa tumor suppressor encoded by ten chromosomes [15], is a hydrophobic, nonsecretory, and transmembrane-free protein mainly located in the cytoplasm (60.9%). The secondary structure of LHPP protein predicted by the SOPMA database mainly contains random crimp (36.67%) and alpha-helix (35.93%) belonging to the NagD superfamily [22]. NetPhos3.1 and PhosphoSite Plus software demonstrate its corresponding structure including phosphorylation, acetylation, and ubiquitination sites [22]. Recently, mounting evidence has indicated that *LHPP* downregulation is highly associated with the poor prognosis of patients with multiple cancers. *LHPP* reduction promoted bladder cancer cell proliferation and growth by mediating the AKT/p65 signaling pathway [18]. In pancreatic cancer, enhanced *LHPP* expression restored PTEN protein and repressed the AKT phosphorylation resulted to inhibition of PaCa (Pancreatic cancer) progression and metastasis [17]. Wang et al. [19] illustrated that *LHPP* could not only repress aggressive phenotype but also trigger autophagy in papillary thyroid cancer. In a previous study [20], *LHPP* expression was significantly lower in colorectal cancer tissues and inversely correlated to tumor severity and worse overall survival. Interestingly, LHPP dysregulation was commonly observed in many cancer types, as LHPP has common characteristics in cancer development. Thus, the biological functions of LHPP in gastrointestinal and esophageal cancers were investigated. Surprisingly, LHPP might have little impact on cell proliferation and metastasis of esophageal cancer (see results in Supplemental Fig. 6).

This research further supported the tumor-suppressor role of *LHPP* in CRC. Overexpressing LHPP protein not only inhibited CRC cells migration and invasion in vitro but also repressed tumor metastasis in vivo*. LHPP* eliminated lung metastatic nodules in nude mice. Consistently, upregulation of E-cadherin and TGF-β1 proteins and downregulation of N-cadherin protein were observed via IHC and western blot analysis in a xenograft model. These results were the opposite in *LHPP* knockdown experiments. Moreover, this study illustrated that upregulating *LHPP* levels inhibited EMT by mediating the classical TGF-β/Smad signaling pathway. Expression of the pivotal regulators in the TGF-β/Smad pathway, including p-Smad3, Smad7, and Jun, led to significant changes, such as a decrease in downstream molecules, such as N-cadherin, MMP2, Snail, Slug, and Twist1. Interestingly, we found variations of downstream molecules were more obvious after blocking *LHPP* expression, suggesting that *LHPP* might have an important role in maintaining the growth and development of normal epithelial cells. Besides, expressions of N-cadherin and Smad4 expressions were extremely weak in the Sw480 cell line. This phenomenon might be caused by tumor heterogeneity.

Generally, TGF-β ligand binding to TGF-β receptors I and II transfers extracellular signal into cell nuclear components via the canonical TGF-β/Smad pathway and non-canonical TGF-β pathways, such as the p38/MAPK pathway, GTP pathway, PI3K/AKT pathway, and NF-κB pathway [23–25]. Therefore, SIS3, the specific inhibitor of p-Smad3 [26, 27], was used to verify the function of *LHPP* in the TGF-β pathway. The changes in EMT-related protein levels were abolished in CRC cells after SIS3 treatment. Transwell assays also suggested that cell numbers in the underlayer did not significantly differ between experimental and NC groups after using SIS3. Thus, the data above uncovered the underlying mechanism of *LHPP* in *CRC* migration and invasion (Fig. 7g). The Eif2 pathway has also attracted authors’ interest after bioinformatics analysis. Previously [28, 29], Eif2 phosphorylation induced CHOP protein levels, resulting in cell apoptosis accompanied by upregulation of Bax protein level and Bcl-2 downregulation. Thus, cell apoptosis and the expression levels of p-Eif2 and Bax proteins were evaluated after LHPP knockdown, but no statistical difference was observed.

The TGF-β superfamily of cytokines encoded by 33 distinct genes, including TGF-β, bone morphogenetic proteins (BMPs), Nodal, inhibins, and growth and differentiation factors (GDFs), is evolutionarily conserved and involved in multiple cellular processes, such as cell proliferation, EMT, and immunosuppression [30, 31]. There are 12 transmembrane kinase receptors subdivided into seven type I (termed activin receptor-like kinases, ALKs) and five type II receptors (TbRII, ActRII, ActRIIB, BMPRII, and AMHRII) [25, 30, 31]_._ The key mediators and signal transmitters of the TGF-β signaling pathway are Smads. The eight Smad proteins are classified into three functional groups: five receptor-regulators Smad 1/5/8 and Smad2/3, one co-Smad protein4 and two inhibitors for negative feedback Smad6/7. Interestingly, in the early process of tumorigenesis, TGF-β, serves as a tumor suppressor, arrests cell cycle, and induces apoptosis via upregulating cyclin-dependent kinase inhibitor (CKIs) and downregulating c-Myc protein [24, 30, 31]. However, in the late stages of cancer, TGF-β is one of the most powerful drivers of cells progression and metastasis through activating EMT process [25, 30, 31]. This study demonstrated *LHPP* could mediate CRC cell EMT by inhibiting the phosphorylation of Smad3 protein and restraining extracellular signal transduction. However, at what stage does LHPP work during tumor progression: precancerosis or advanced cancer? Thus, the next study will focus on the further mechanism of LHPP biological functions during different cancer stages.

Certainly, there are some limitations to this research. First, the upstream molecular mechanism of *LHPP* downregulation in CRC was not explored. Tian et al. [21] illustrated that miR-365-5p and miR-765 could target *LHPP* expression in AFP-negative hepatocellular carcinoma (AFP-HCC). Therefore, blocking of LHPP protein levels in CRC tissues might be correlated to miRNA or CpG hypermethylation [32]. Second, the interaction between Smad3 and LHPP proteins remains unclear. Thus, further investigation, such as co-immunoprecipitation assay, into the reciprocal relationship between *LHPP* and Smad3 should be performed.

In conclusion, functional studies indicated that LHPP is a tumor suppressor that inhibits CRC proliferation and metastasis in vivo and in vitro. Mechanistically, LHPP specifically inactivates Smad3 phosphorylation—the key mediator in the TGF-β/Smad signaling pathway. These novel results provide new perspectives for CRC treatment and developing therapeutic targets.

## Materials and methods

### Immunohistochemistry (IHC)

IHC was performed as described [20] previously. Briefly, heterologous tumor tissues from nude mice were fixed in formaldehyde and embedded in paraffin. Sections were incubated with rabbit polyclonal antibodies overnight at 4 °C. Two independent investigators evaluated staining blindly.

### Cell lines and culture conditions

Human CRC cell lines (HT-29, Sw480, and Caco2) were purchased from the Cell Bank of the Chinese Academy of Sciences (Shanghai China). Sw480 and Caco2 cells were cultured in complete RPMI 1640 medium (Hyclone, USA). HT-29 cells were cultivated in complete Dulbecco’s modified Eagle’s medium (DMEM Hyclone, USA). The medium was supplemented with 10% fetal bovine serum (FBS Gibco) and 1% penicillin–streptomycin mixture. Cells were maintained at 37 °C in a humidified incubator with 5% CO_2_.

### Immunofluorescence assay (IF)

IF assay was performed as described [20] previously. Briefly, cells (1 × 10^4^) were seeded on slides in a 24-well plate and cultured in an incubator for 24 h. Ice-cold 4% paraformaldehyde was used to fix cancer cells for about 20 min and 0.5% Triton X-100/ phosphate buffer saline (PBS) was used to permeabilize cells for 30 min. Cells were incubated with primary antibody (p-Smad3 dilution 1:100, Catalog no:ab52903; Abcam) overnight at 4 °C after blocking with bovine albumin (BSA) for about 20 min. Goat anti-rabbit IgG/RBITC (Bioss, BS-0295G, Beijing, China) was added for about 1 h. Cell nuclei were counterstained with 4,6-diamidino-2-phenylindole. Cells were observed under a fluorescence microscope.

### Cell transfection

The cell transfection protocol was described before [20]. Briefly, a total of 20–30% Sw480 and Caco2 cells were stably transfected with *LHPP* lentiviruses (LV) or negative control LVs (NC) according to the manufacturer’s protocol (viral volume = MOI× cell numbers/viral titers; GeneChem Co., Ltd, Shanghai, China; MOI = 20, cell numbers: 1–5 × 10^5^, viral titers: 4 × 10^8^). Lentiviral vectors used the GV208 system combined with the cytomegalovirus promoter-driven puromycin gene and green fluorescent protein. The GV248 RNA interference (RNAi) system (GeneChem, Shanghai, China) was used to impede *LHPP* expression. The target sequence of *LHPP* was 5’- GAGCAAGGCCUGCGACCAUTTAUGGUCGCAGGCCUUGCUCTT-3’. The negative sequence was 5’- UUCUCCGAACGUGUCACGUTT-3’. HT-29 and Sw480 cell lines were stably transfected with *LHPP-RNAi* lentiviruses (viral volume = MOI× cell numbers/viral titers; MOI = 20, cell numbers: 1–5 × 10^5^, viral titers: 2 × 10^9^).

### Nuclear protein extraction

The Nuclear Protein Extraction kit (Abcam, USA, ab113474) was used to extract nuclear proteins from cells. Cells were cultured to 60–70% confluence, and we scraped cells into a 15-ml conical tube after adding 1 ml fresh PBS per 20 cm^2^ area. Cells were centrifuged for 5 min at 1000 rpm, and the supernatant was discarded. Next, cells were resuspended in 100 μl of 1×preextraction buffer per 10^6^ cells and incubated on ice for 10 min. The prepared extract was centrifuged for about 1 min at 12,000 rpm after vigorous vortexing. Notably, it is extremely important to remove the cytoplasmic extract from the nuclear preparation. Then, two volumes of extraction buffer containing PMSF were added to the nuclear pellet (about 10 μl per 10^6^ cells) and incubated for 15 min with vortexing (5–15 s) every 3 min for about 0.5 h. Finally, the suspension was centrifuged again for 10 min at 12,000 rpm at 4˚C and the supernatant was transferred into a new tube. Protein concentrations were determined by using a BCA Protein Assay kit (cat. no. PA115‑01; Tiangen Biotech Co. Ltd.).

### Western blot analysis

The western blot was performed as described [20] previously. Total proteins were isolated from cells or tissues by using radioimmunoprecipitation assay buffer with protease inhibitors. Equal amounts of protein (20–30 μg) were separated by 10% or 12% sodium dodecyl sulfate-polyacrylamide gel electrophoresis and then transferred to polyvinylidene difluoride membranes. Membranes were incubated with primary antibodies overnight at 4 °C after blocking with 5% milk, followed by incubation with a secondary antibody (1:5000). Image J software (National Institutes of Health) was used to examine the gray values of each primary antibody and glyceraldehyde 3-phosphate dehydrogenase (GAPDH). The ratio of gray values (primary antibody/GAPDH) was calculated using GraphPad Prism 6 software (Graph Pad Software, Inc.). The other antibodies are shown in the Supplemental Table.

### RNA extraction, reverse transcription, and real-time PCR (RT-PCR)

The total RNA was extracted from cells using the Fastagen200 Kit (Shanghai China, Cat:220010) according to the manufacturer’s protocol. cDNAs were synthesized with PrimeScript™ RT Master Mix (Takara Biotechnology, China). Quantitative RT-PCR procedures were performed by using the SYBR Green PCR Kit (Takara Biotechnology, China) in three independent experiments. The *LHPP* expression was calculated using the 2^−ΔΔCq^ method. The housekeeping gene was *GAPDH*. The following primers and procedures were described before [20].

### Cell viability assay

Cells were seeded (2000–3000 per well) in a 96-well plate and cultured for 7 days. Next, a 10 μl CCK-8 solution (E1CK-000208, Nanjing, China) was added to each well, and cells were incubated for 2 h under aseptic conditions in a 5% CO_2_ incubator at 37 °C. The spectrophotometric value of each sample was measured at 450 nm.

### Colony-formation analysis

Cells were (1000–2000 per well) plated in culture plates for 3–4 weeks at 37 °C in a humidified environment with 5% CO_2_ and stained with crystal violet staining solution(1%) for 30 min. The stained colonies were imaged using a camera and counted using a microscope.

### Cell cycle analysis

Cells (1–2 × 10^5^) were seeded in 6-well plates and cultured for 24 h. Cells were digested and collected in a new EP tube and fixed them with cold ethanol at 4 °C overnight. After this, 500 µl propidium iodide (PI) and RNase A (1:9) were applied to incubate cells in the darkness (Cat. no. KGA512, KeyGen Biotech, Nanjing China). The results were analyzed by using a FACSCalibur flow cytometer (BD Bioscience). The percentage of different cell cycles was calculated using Graphpad Prism 6 software (GraphPad Software, Inc.).

### Cell apoptosis assay

Cells (5–10 × 10^4^) were plated in six-well plates to culture 24–48 h after transfection with LVs. Cells were digested using 0.25% trypsin without EDTA and collected cells into new 15-ml tubes. Subsequently, cells were stained with *7AAD* for cell nucleus and *PE* for cytomembrane (Cat. no. KGA1017, KeyGen Biotech, Nanjing China). Cell apoptosis was examined using FACSCalibur flow cytometer (BD Bioscience).

### Wound-healing assay

Cells (1 × 10^5^) were seeded in six-well plates and cultured to >95% confluence. Wounds were scratched using a 10-μl plastic pipette tip. After PBS wash, cells were cultured with 1–2% serum. Wounded areas were photographed by phase-contrast microscopy at 0 h, 24, 48 h, respectively.

### Cell migration assay

Cells (2 × 10^4^) were seeded with serum-free medium (200 μl) in a Transwell migration chamber (Corning, USA) with an 8-μm pore membrane on the bottom. The chamber was inserted in a well of a 24-well plate containing 10% FBS media (500 μl DMEM or RPMI 1640). Cells were incubated at 37 °C in a humidified environment with 5% CO_2_ for 12–24 h, and then stained with crystal violet and detected under a microscope after being fixing with 4% paraformaldehyde. A microscope was used to image migrating cells and cell numbers were counted under five independent visual fields. Statistical analysis was done using Graphpad Prism 6 Software (GraphPad Software, Inc.).

### Cell invasion assay

Transwell invasion cells (1 × 10^5^) were plated on top of a 24-well Corning 8-μm pore membrane with a serum-free medium. The matrix gel was added on top of the Transwell chamber (BD, USA, dilution 1:8, 50–80 μl, diluted with medium). After 24–48 h, invading cells were washed with PBS, fixed with 4% paraformaldehyde, and stained with crystal violet. Bright-field microscopy was used to count invading cell numbers. A microscope was used to image migrating cells, and cell numbers were counted under five independent visual fields. Statistical analysis was calculated using Graphpad Prism 6 Software (GraphPad Software, Inc.).

### Microarray analysis

Total RNA was isolated from Caco2 CRC cells after transfection with *OE-LHPP* LVs and the NC group for 72 h (three replicates each group). RNA samples were analyzed by microarray expression profiling using the Affymetrix Human Gene 1.0 ST platform (*Affymetrix*) according to the manufacturer’s instructions (GeneChem, Shanghai, China). Genes with ≥1.2-fold change between two groups were identified as differentially expressed genes (DEGs). DEGs were analyzed using Ingenuity Pathway Analysis (IPA) commercial software

### Inhibitor of the TGF-β/Smad signaling pathway

To determine whether *LHPP* mediated the EMT process via the TGF-β signaling pathway. Experimental and negative control cells were treated with TGF-β1 (Abcam, ab50036, 5 ng/ml) after being serum starvation for 24 h. TGF-β1 was reconstituted in 10 mM citric acid (50 μg/ml) for storage and further diluted in PBS (5 ng/ml). Subsequently, migration and invasion abilities were determined using Transwell and wound-healing assays. Next, the TGF-β/Smad inhibitor-SIS3-(MCE, USA, HY-13013) was added to cancer cells. Cell migration and invasion experiments were performed once again after cancer cells were treated with SIS3. Western blot analysis was performed to evaluate the expression of total and nuclear proteins.

### Xenograft assays and lung metastasis models

All animal experiments were performed in accordance with the institutional guidelines and were approved by the Laboratory Animal Center of Xi’an Jiaotong University. Four-week-old female BALB/c-nude mice were purchased from Xian Jiaotong University Animal laboratory for subcutaneous xenograft experiments. Caco2 cells (2–4 × 10^6^/200 μl) with *LHPP-*overexpression LVs and blank vectors were injected subcutaneously into mice (six mice per group). Tumor size was measured using caliper every 3 days and calculated using the formula: volume = length × (width^2^)/2. Lung metastasis models were built by injecting Caco2 cells (1–2 × 10^6^/200 μl) into tail veins (5 mice per group). After 7–8 weeks, mice were killed by cervical dislocation. Humane endpoints [33, 34] were as followed (i) tumor ulceration showed no stabilization within 7 days of treatment; (ii) ulcerated tumor was actively bleeding; (iii) ulcerated tumor showed visible signs of infection; (iv) animals showed discomfort associated with tumor ulceration such as biting/scratching; and (v) tumor size did not exceed 20 mm (2.0 cm) in mice (IACUC Guideline: Tumor Induction in mice and rats). Tumors and lungs were isolated from mice. Tumor tissues were fixed in 4% paraformaldehyde and cut into 10-μm sections for IHC analysis.

### Statistical analysis

All statistical data were calculated using GraphPad Prism 6 software. The experimental results were repeated three times and expressed as the mean ± standard error. A two-tailed Student’s *t* test was used to analyze the statistical significance between different groups. One- or two-way analysis of variance (ANOVA) followed by Bonferroni’s multiple comparison test, was performed to test the difference between multiple groups. *P* < 0.05 was regarded as statistically significant.

## Supplementary information

cddiscovery author contribution form

Revised Supplemental figure legends

Revised Supplemental Figure 1

Supplemental figure2

Revised figure 3

Revised Supplemental figure 4

Revised Supplemental figure5

Revised Supplemental Figure 6

Supplemental table

## Data Availability

The data that support the findings of this study are available from the corresponding author upon reasonable request.
