# Supplementary material for: LHPP suppresses colorectal cancer cell migration and invasion in vitro and in vivo by inhibiting Smad3 phosphorylation in the TGF-β pathway
Source: Cell Death Discov. 2021 Oct 4;7:273. doi: 10.1038/s41420-021-00657-z (PMC8490460; doi:10.1038/s41420-021-00657-z)
Supplement: Supplementary file 1 — cddiscovery author contribution form [file 41420_2021_657_MOESM1_ESM.pdf]

DECLARATION OF CONTRIBUTIONS TO ARTICLE

ADMC

Manuscript Number: CDDISCOVERY-21-1336R2

Journal Name: *Cell Death Discovery* (the 'Journal')

Proposed Title of the Contribution: LHPP suppresses colorectal cancer cell migration and invasion in vitro and in vivo by inhibiting Smad3 phosphorylation in the TGF-β pathway (the 'Contribution')

Author(s): Bin Hou, Wenhan Li, Peng Xia, Fengyu Zhao, Zhao Liu, Qingnuo Zeng, Shilong Wang, Dongmin Chang (the 'Authors')

For all *CDDiscovery* articles, each person named as an author in the published version must be able to show he or she has contributed substantially to the article.

Authorship credit should be based on 1) substantial contributions to conception and design, acquisition of data, or analysis and interpretation of data; 2) drafting the article or revising it critically for important intellectual content; and 3) final approval of the version to be published. Authors should meet conditions 1, 2 and 3.

Any person who cannot be shown to have made a substantial contribution to the article cannot be listed as an author in the final version. The name of any person who is deemed to have made a minor contribution can, however, appear in the Acknowledgments section of the article.

Please complete the table below to indicate the contributions of all named authors to the manuscript.

Author Full Name:	Specification of Contribution to the Manuscript:
Bin Hou	Conception and design, Doing experiments, Writing manuscript, Acquisition of data
Wenhan Li	Conception and design, Analysis of data
Peng Xia	Acquisition of data, Revise and edit manuscript
Fengyu Zhao	Doing experiments, Writing manuscript, revise manuscript
Zhao Liu	Doing expreiments, Providing experimental materials
Qingnuo Zeng	Acquisition of data, Analysis and interpretation of data
Shilong Wang	Doing experiments, Analysis of data
Dongmin Chang	Funding support, Conception and design, Revise and edit manuscript

Please complete the table below to indicate the contributions of all named authors to the figures.

Figure 1: Bin Hou, Zhao Liu, Fengyu Zhao, Peng Xia, Dongmin Chang

Figure 2: Bin Hou, ShiLong Wang, Qingnuo Zeng

Figure 3: Bin Hou, Zhao Liu, Fengyu Zhao, Dongmin Chang

Figure 4: Bin Hou, Shilong Wang, Qingnuo Zeng

Figure 5: Bin Hou, Peng Xia, Wenhan Li, Dongmin Chang

Figure 6: Bin Hou, Dongmin Chang, Fengyu Zhao

Signed for and on behalf of the Author(s): HB, LWH, XP, ZFY LZ, ZQN, WSL, CDM

Print Name: Bin H,Wenhan L,Peng X,Fengyu Z,Zhao L,Qingnuo Z, Shilong W,Dongmin C

Date: 2021-09-12
