# Supplementary material for: LHPP suppresses colorectal cancer cell migration and invasion in vitro and in vivo by inhibiting Smad3 phosphorylation in the TGF-β pathway
Source: Cell Death Discov. 2021 Oct 4;7:273. doi: 10.1038/s41420-021-00657-z (PMC8490460; doi:10.1038/s41420-021-00657-z)
Supplement: Supplementary file 2 — Revised Supplemental figure legends [file 41420_2021_657_MOESM2_ESM.doc]

Supplemental Figure Legends

*Supplemental figure1 (a) Positive Caco2 cells expressed green fluorescent protein, the transfection efficiency was >80%; (b) Expression level of LHPP protein in Caco2 cell line following transfection lentiviruses 72 hours by using Western blot; (c) Relative mRNA expression of LHPP in Caco2 cell line was evaluated using RT-qPCR and GraphPad Prism6 software; (d) Relative mRNA expression of LHPP in Sw480 cell line was determined via using RT-qPCR and GraphPad Prism6 software; (e) The result in Sw480 cell line was shown following transfection knockdown lentiviruses. ***P < 0.001, ****P < 0.0001.*

*Supplemental figure2 (a,c,e) Growth and proliferation of Caco2 were inhibited by over-expressing LHPP protein. The CCK-8 assay demonstrated that viability of Caco2 was reduced at 3, 5 and 7days after transfection with lentiviruses. Clone numbers in Caco2 negative group were much more than clone numbers in OE-LHPP group; (b,d, f) Knockdown of LHPP protein could promote viability of Sw480 cells at 3, 5 and 7 days following stably transfection. Meanwhile, Clone numbers were increased after culturing 4 weeks in Sh-LHPP group. **P < 0.01,***P < 0.001, ****P < 0.0001.*

*Supplemental figure3 (a) Up-regulating expression of LHPP could arrest Caco2 cells in G_0_/G_1_ phase after PI staining; (b) The statistical differences of G_0_/G_1_, S and M phases were calculated via using Graphpad Prism 6 software; (c) Effect of LHPP down-regulation on cell cycle distribution, typical images of DNA content examined by flow cytometric analysis following PI staining; (d) Percentages of different cell cycle phases were determined by using Graphpad Prism 6 software from three individual experiments; (e) Proteins related to cell cycle, including P53, CyclinD1/CDK4, PCNA and NME1, were evaluated through Western blot; (f) Relative expression levels of proteins were analyzed using the Image J and Graphpad Prism 6 software in OE-LHPP group; (g) Relative expression levels of proteins were calculated using the Image J and Graphpad Prism 6 software after depletion of LHPP expression. **P < 0.01,***P < 0.001, ****P < 0.0001.*

*Supplemental figure4 Effect of LHPP knockdown on colorectal cancer cell apoptosis. Representative profiles of Sw480 cells (a) and HT-29 cells (c) apoptosis tested by using 7 AAD/PE staining and FACS analysis; Apoptotic cell rates were analyzed on Sw480 cells (b) and HT-29 (d). There was no obvious difference between LHPP-depletion group and their negative control group (Sw480 P =0.115 and HT-29 P =0.075 respectively); (e, f) Cell apoptosis-related proteins, like Bax, Caspase3, Eif2 and p-Eif2, were evaluated using Western blot in different cell lines, No significant difference was observed. Ns: no significance.*

*Supplemental figure5 Results of different SIS3 concentration on expression levels of p-Smad3 and EMT-related proteins. Firstly, HT-29 (a), Caco2 (b) and Sw480 (c) cells were pre-treated with different concentration of SIS3 (MCE, USA,1~10ng/ml) for 4-6 hours. Subsequently, we added TGF-β1 (5 ng/ml) to medium for about 24 hours following starving colorectal cells one day. Next, Western blot was performed to evaluate expression of relative proteins, including p-Smad3, E-cadherin, N-cadherin, Snail, Twist1.*

*Supplemental figure6 Biological functions of LHPP in esophagus cancer. (a) Protein expression of LHPP was tested in esophagus cancer tissues and their counterparts. Interestingly, down-regulation of LHPP protein level was not obvious in cancer tissues. N: normal tissue; C: Cancer tissue; (b) Basic expression level of LHPP was examined in esophagus cancer cell lines, including TE-1, KYSE150 and Eca109; (c, d) Transwell assay was performed to determine migration and invasion abilities of KYSE-150 cells with silencing LHPP expression and TE-1 cells with enhancing LHPP expression, respectively. There was no obviously significant difference between experimental group and control group; (e, f) Protein levels of downstream molecules were examined following LHPP over-expression and down-expression in TE-1 and KYSE150 cells by using Western blot. Most protein levels did not have obvious change, except Bcl-2, between two groups.*
