# Supplementary figures and images for: LHPP suppresses colorectal cancer cell migration and invasion in vitro and in vivo by inhibiting Smad3 phosphorylation in the TGF-β pathway
Source: Cell Death Discov. 2021 Oct 4;7:273. doi: 10.1038/s41420-021-00657-z (PMC8490460; doi:10.1038/s41420-021-00657-z)

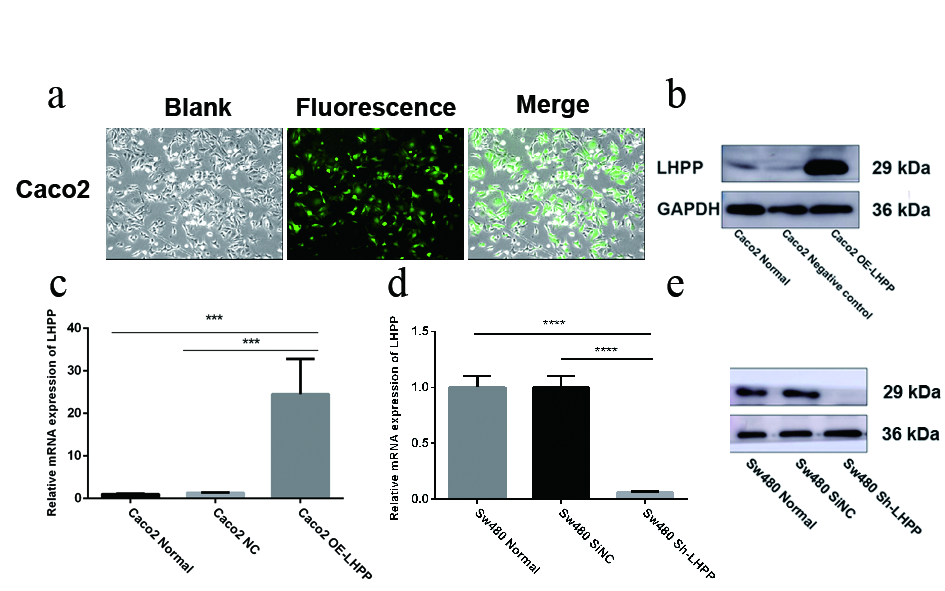

Supplement: Supplementary file 3 — Revised Supplemental Figure 1 [file 41420_2021_657_MOESM3_ESM.tif]

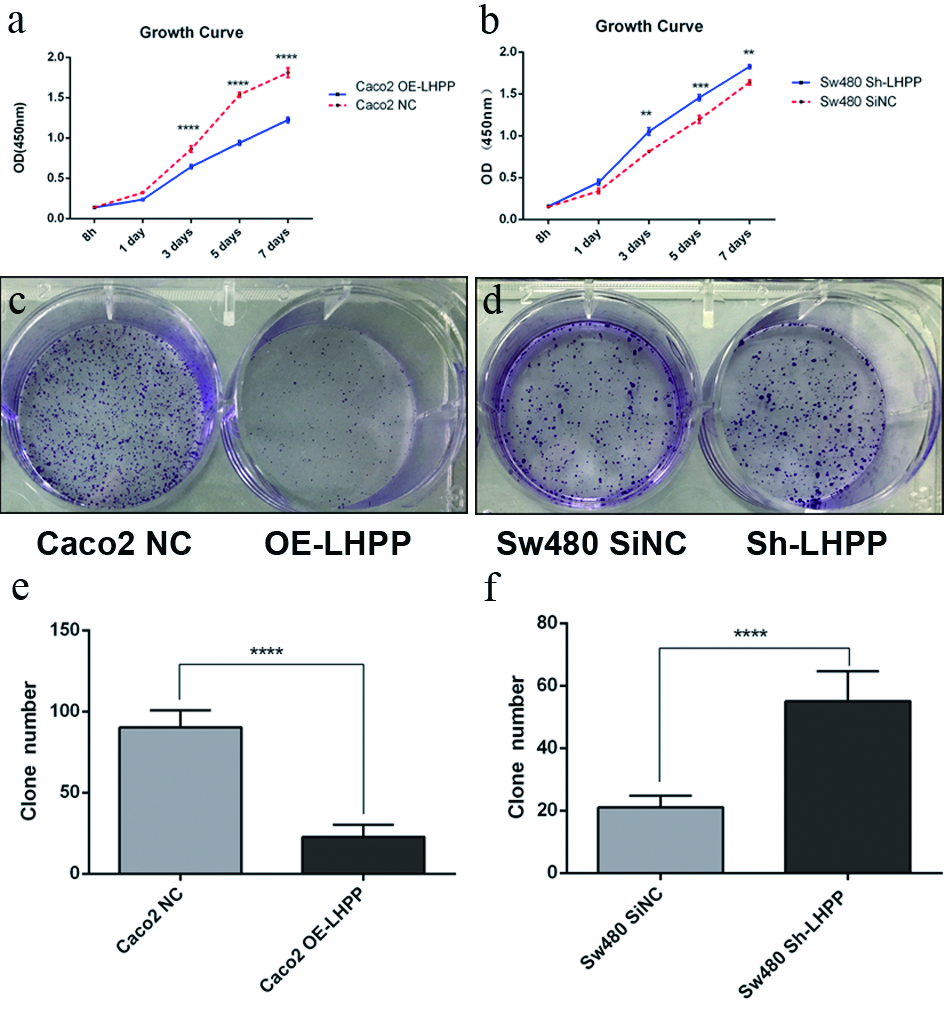

Supplement: Supplementary file 4 — Supplemental figure2 [file 41420_2021_657_MOESM4_ESM.tif]

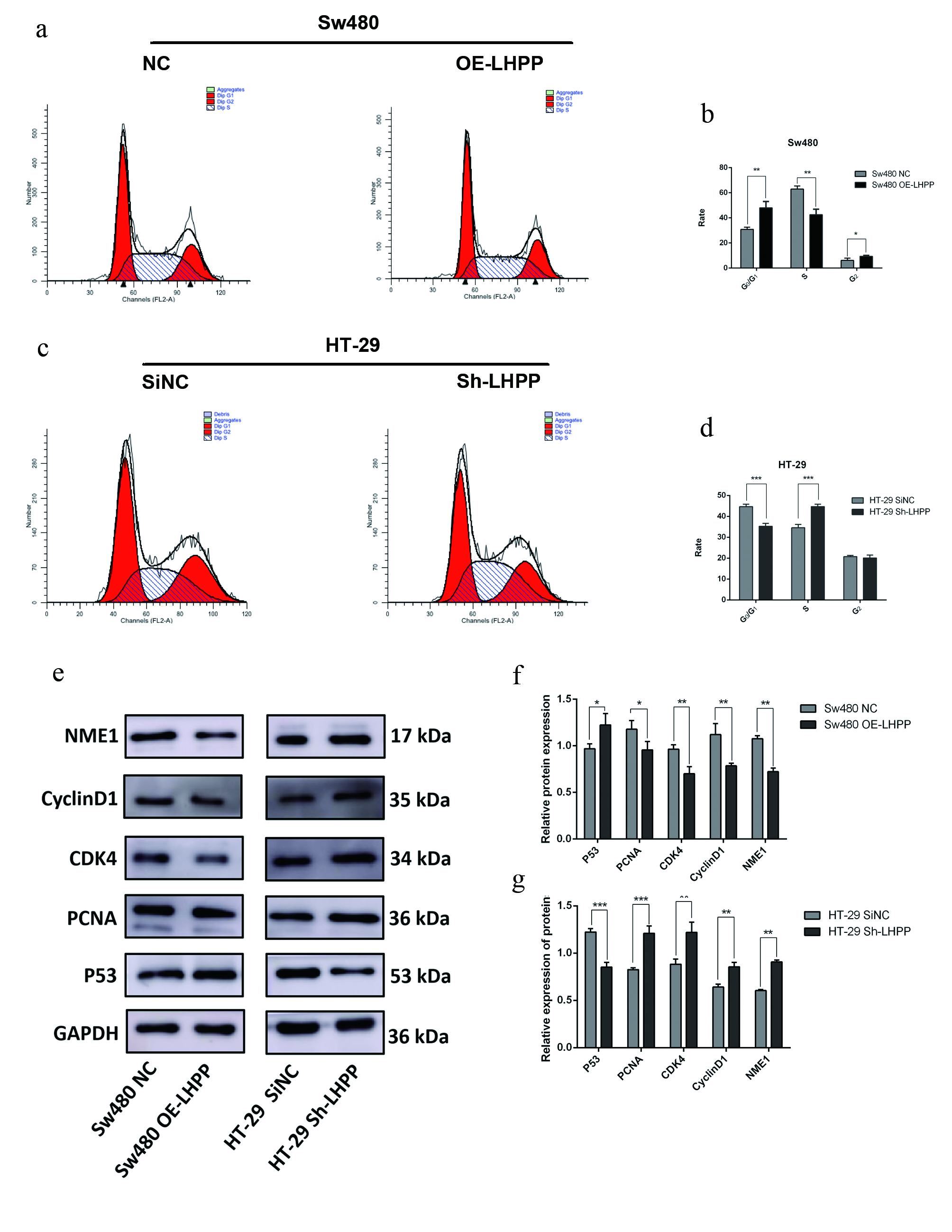

Supplement: Supplementary file 5 — Revised figure 3 [file 41420_2021_657_MOESM5_ESM.tif]

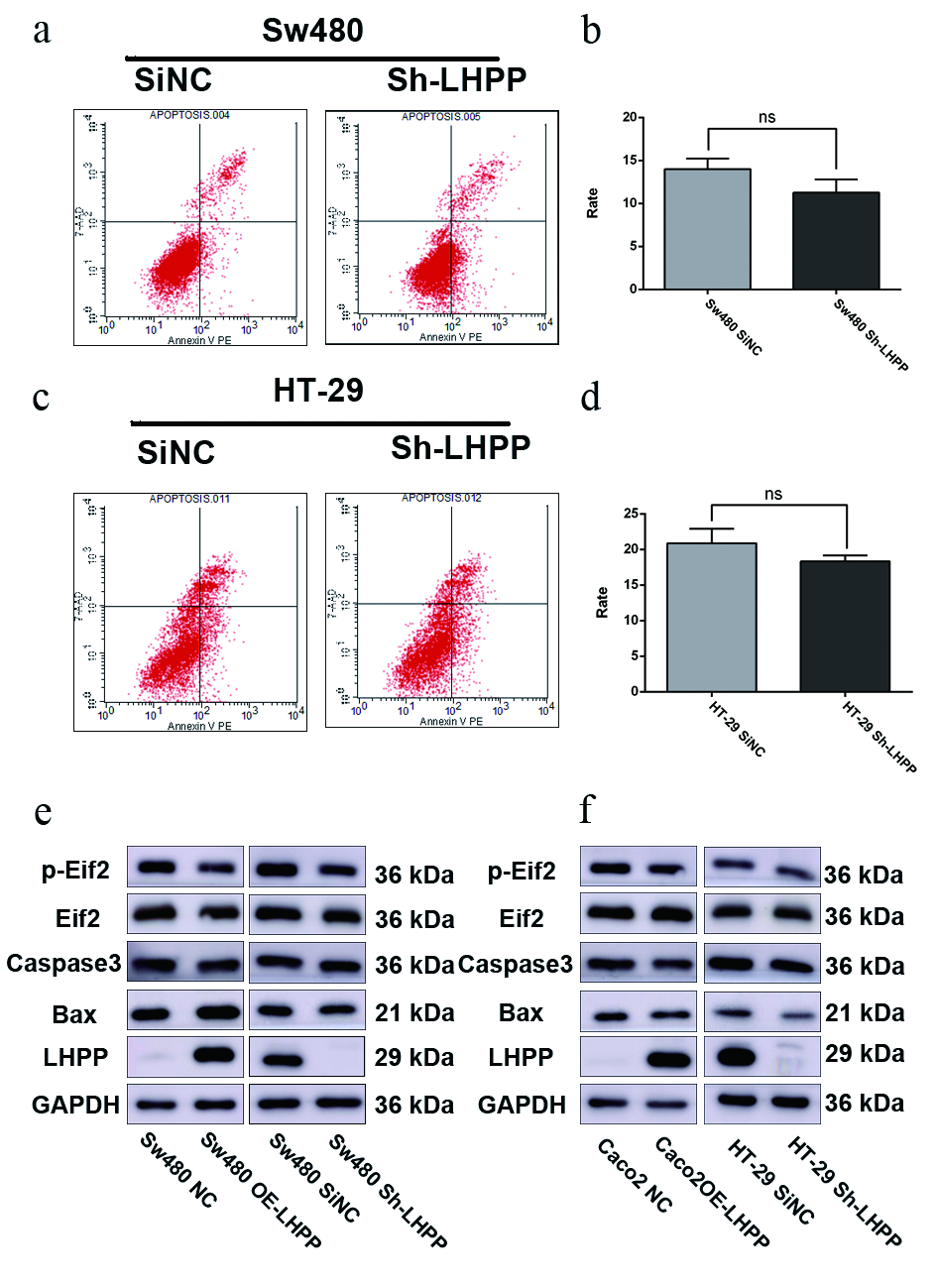

Supplement: Supplementary file 6 — Revised Supplemental figure 4 [file 41420_2021_657_MOESM6_ESM.tif]

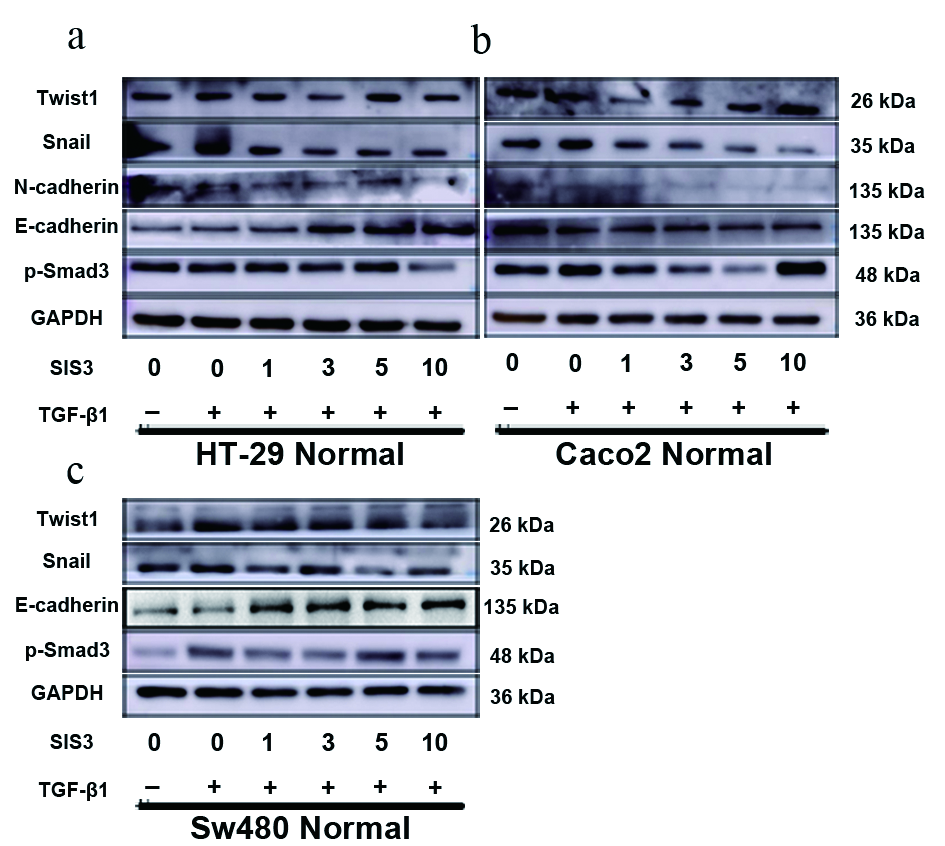

Supplement: Supplementary file 7 — Revised Supplemental figure5 [file 41420_2021_657_MOESM7_ESM.tif]

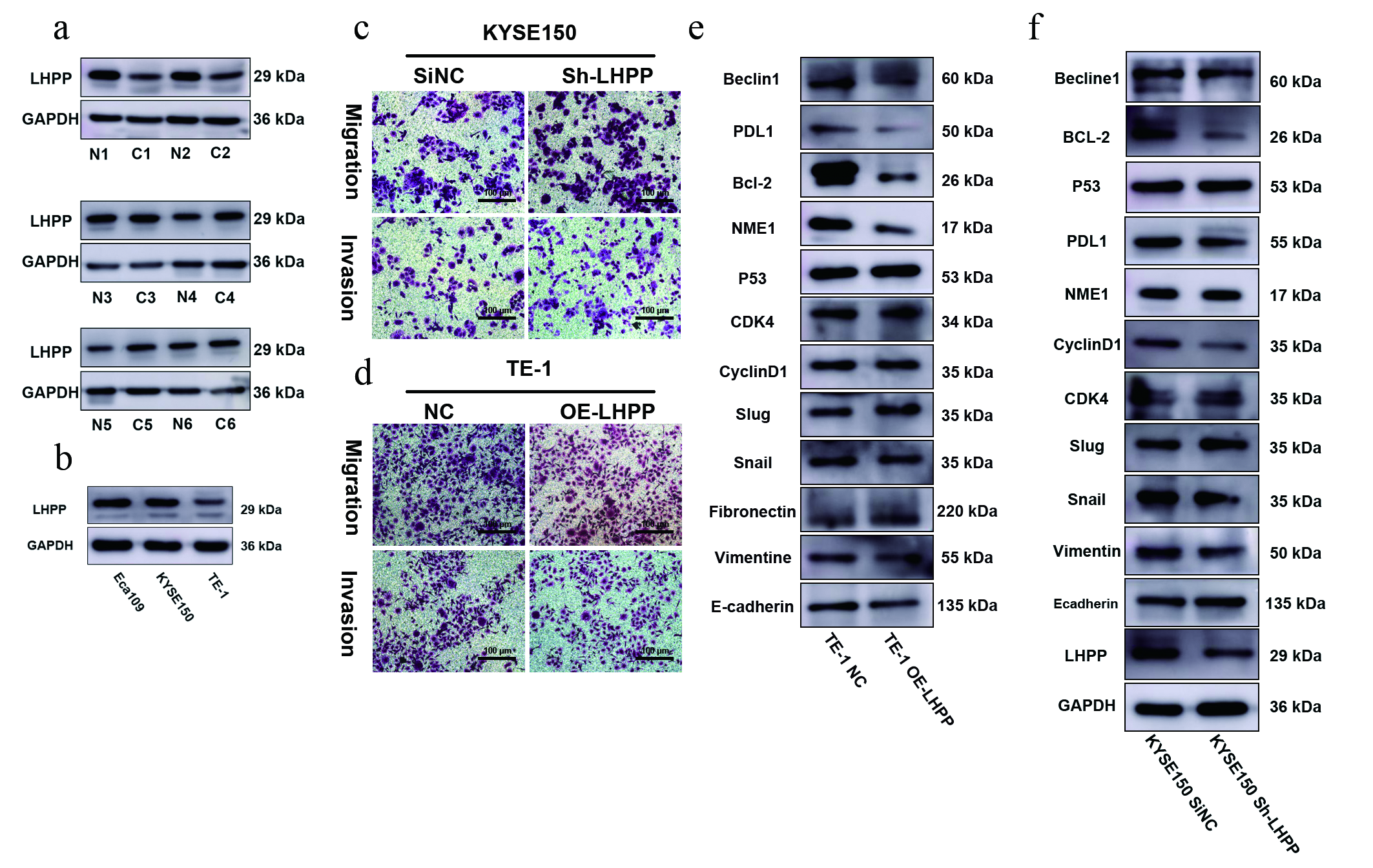

Supplement: Supplementary file 8 — Revised Supplemental Figure 6 [file 41420_2021_657_MOESM8_ESM.tif]
