# Supplementary material for: LHPP suppresses colorectal cancer cell migration and invasion in vitro and in vivo by inhibiting Smad3 phosphorylation in the TGF-β pathway
Source: Cell Death Discov. 2021 Oct 4;7:273. doi: 10.1038/s41420-021-00657-z (PMC8490460; doi:10.1038/s41420-021-00657-z)
Supplement: Supplementary file 9 — Supplemental table [file 41420_2021_657_MOESM9_ESM.doc]

**Supplementary table. Primary antibodies used in western blot analysis.**

| **Name** | **No.** | **Dilutions** | **Vendor** |
| --- | --- | --- | --- |
| Bax | 50599-12-Ig | 1:2000 | Proteintech |
| Caspase-3 | 66470-2-Ig | 1:1500 | Proteintech |
| CDK4 | 49132 | 1:2000 | SAB |
| Cyclin D1 | WL01435a | 1:1000 | Wanleibio |
| E-cadherin | 3195T | 1:1500 | CST |
| Eif2 | 11233-1-AP | 1:1000 | Proteintech |
| p-Eif2 | Ab32157 | 1:1000 | Abcam |
| Fos | 66590-1-Ig | 1:1000 | Proteintech |
| GAPDH | 10494-1-AP | 1:10000 | Proteintech |
| Jun | 24909-1-AP | 1:1000 | Proteintech |
| LHPP | 15759-1-AP | 1:1000 | Proteintech |
| MMP2 | WL03224 | 1:1000 | Wanleibio |
| N-cadherin | 13116T | 1:1000 | CST |
| NME1 | 11086-2-AP | 1:1000 | Proteintech |
| PCNA | 10205-2-AP | 1:1000 | Proteintech |
| P53 | 10442-1-AP | 1:1000 | Proteintech |
| Slug | WL01508 | 1:500 | Wanleibio |
| Smad3 | 25494-1-AP | 1:500 | Proteintech |
| p-Smad3(S423+425) | Ab52903 | 1:1000 | Abcam |
| Smad4 | 10231-1-AP | 1:500 | Proteintech |
| Smad7 | 25840-1-AP | 1:500 | Proteintech |
| Snail | WL01863 | 1:500 | Wanleibio |
| Twist1 | WL00997 | 1:500 | Wanleibio |
